# Colony: a framework for reproducible and easy-to-use data analysis pipelines for biomedical research with singularity containers

**DOI:** 10.1093/bioadv/vbaf304

**Published:** 2025-11-26

**Authors:** Sebastian Eschner, Mohammad Alabdullah, Martin Dugas

**Affiliations:** Institute of Medical Informatics, Heidelberg University Hospital, 69120 Heidelberg, Germany; Department of Dermatology, Heidelberg University Hospital, 69120 Heidelberg, Germany; Institute of Medical Informatics, Heidelberg University Hospital, 69120 Heidelberg, Germany

## Abstract

**Summary:**

Bioinformatics pipelines should meet the FAIR criteria to enable reproducible analysis. FAIR describes four key requirements for reproducible research: findability, accessibility, interoperability and reusability. Software containers such as Singularity are widely used tools that facilitate the reuse of software across different computing environments. However, many biologists and other researchers find command line tools such as Singularity unfamiliar and do not feel productive when using software via the command line. We present a graphical user interface that allows biologists without programming experience to interact with containerized software. We evaluate the feasibility of our approach with software used at the TRR156.

**Availability and implementation:**

Colony can be freely downloaded on its project page: https://clipc-jpg.github.io/ColonyWebsite/. The Colony launcher’s code is MIT-licensed and freely available at: https://github.com/clipc-jpg/Colony. All related assets can be found at: https://doi.org/10.7910/DVN/Z3OTWY.

## 1 Introduction

Biomedical research uses a wide range of technologies and mathematical methods to analyze data. The proliferation and diversity of software tools in biomedicine has led to widely incompatible usage patterns. There are also problems with software compatibility, especially when significant time has passed between publication and attempted replication. Often, information about how software was used turns out to be incomplete. These circumstances lead to a lack of reproducibility: For the reasons mentioned above, it is time-consuming and often impossible to directly recreate the results of biomedical publications. And it is no less difficult to apply software pipelines from previous studies to new datasets and questions. A common name for this problem is “reproducibility crisis” (Baker 2016). Common approaches include package managers (AnacondaInc 2015, R Core Team 2021), workflow managers (Wratten *et al.* 2021), proprietary end-to-end software products or web services (OxfordNanoporeTechnologies 2024), and containerization (Merkel 2014, Kurtzer *et al.* 2017). Containerization means that the software environment, such as the operating system and the programs installed on it, are isolated from the host operating system. This minimizes compatibility problems and dependency conflicts. We want to solve aforementioned problems with a new containerization-based approach that conserves preexisting bioinformatical software in a way such that their use becomes uniform and every component is portable. We aim to make the FAIR principles (Wilkinson *et al.* 2016) applicable to software with the same rigor as is being done to raw data, thereby addressing challenges highlighted by the FAIR4RS principles (Barker *et al.* 2022). In our framework, containerization turns software into a portable, version-stable commodity, reducing the risk of incompatibilities and obsolescence.

## 2 Methods

As part of the INF subproject in the collaborative research center SFB-TRR156 (Enk 2023) we first performed unstructured interviews with researchers from other subprojects and identified the following requirements:

1. The tool needs to enable researchers to perform fully reproducible analyses on RNA-seq data.
2. Common and simple tasks have to be performed reliably and require no more than a few steps only.
3. Operating the tool itself should not require any training.
4. The tool should be operable either on a laptop or, if that is not feasible, on a remote High-Performance Computing Cluster (HPC).

### 2.1 Software architecture

**Figure 1.**
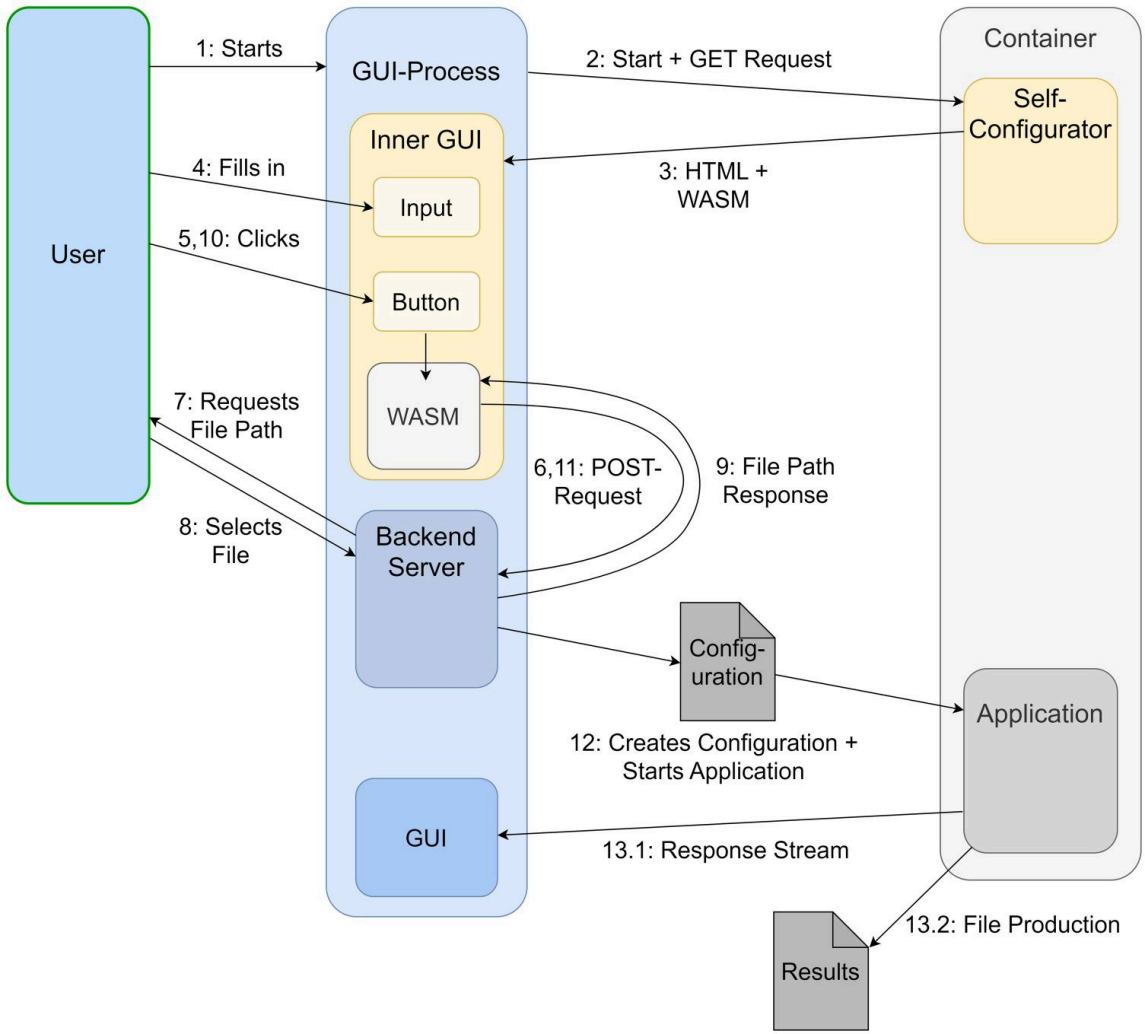
A Diagram of GUI-container interactions during a typical use case. With few ubiquitous tools, a native-like graphical app that resides inside a Singularity container can be realized. The GUI itself remains mostly agnostic to how individual apps operate. A two-step procedure of data production ensures reproducibility.

Our framework aims to perform data analyses for biomedical publications in a necessarily reproducible way. The core design principle is that all user interactions result in a single configuration file first. This configuration file completely defines the workflow a user intends to run and all resulting outputs. Furthermore, the collection of all software necessary to produce publication data from raw measurements, is a singular file as well. This is achieved through Sylab’s SingularityCE containerization technology. Core functionality—i.e. executing the workflow and other workflow-specific tasks—is stored inside and performed by the containers themselves. This functionality is exposed externally through so-called “apps” in the container. Apps are a Singularity concept used when building a container from a definition file. They are specific sections that each give it a separate bash script as entry point and Singularity can run each one individually. This feature supports researchers in creating configuration files through built-in web pages, served by an app conventionally named “self-configurator.” These configuration files are JSON files that serve as a replacement for command-line arguments. They contain all necessary parameters to run a container’s workflow calculations. We design a GUI that informs the user about the availability of all apps and can execute them as per the user’s request. As outlined in Fig. 1, this GUI supports self-configurator apps via its own REST-API endpoints. They make selected native functionality available to these apps, thus enabling the container to execute native-like graphical user interfaces while requiring minimal adaptation from the containerized software. These web interfaces are flexible in implementation (HTML, Rust, etc.) and are not automatically generated, allowing any container-compatible interface to be used while maintaining reproducibility. A user may create a configuration file from such an app or select a preexisting one and then have the GUI execute the analysis described therein locally. However, this launcher GUI is not a prerequisite for using workflow containers. The self-configurator and workflow can be used manually with Singularity and a web browser, making the workflows cross-platform usable.

### 2.2 Implementation of the graphical user interface

The launcher graphical user interface is a binary written in the Rust programming language. It uses the Dioxus library that renders web pages as a desktop app in the native WebView renderers that are available on Windows, MacOS and Linux. Currently, the launcher GUI is only supported on Windows. Extending the launcher to Linux and macOS is technically feasible and will be considered in future releases.

### 2.3 Implementation of containers

We demonstrate our approach by adapting the wf-transcriptomes pipeline from EPI2MELabs, used at the TRR156 to analyze Oxford Nanopore sequencing data. While wf-transcriptomes is a Nextflow workflow, we could not guarantee that all of its runtime requirements would prove compatible with Singularity’s immutable file system. We ensured full reproducibility and reimplemented the workflow in Julia and added a GUI in Rust/Dioxus for configuration. This procedure prioritizes immutability, although in principle a Nextflow run during containerization could capture these requirements directly. To illustrate that Colony is not limited to a single reimplementation, we additionally provide a minimal R-based container on the project page as a proof-of-concept of how simpler tools can be integrated with comparatively little effort.

## 3 Results

**Figure 2.**
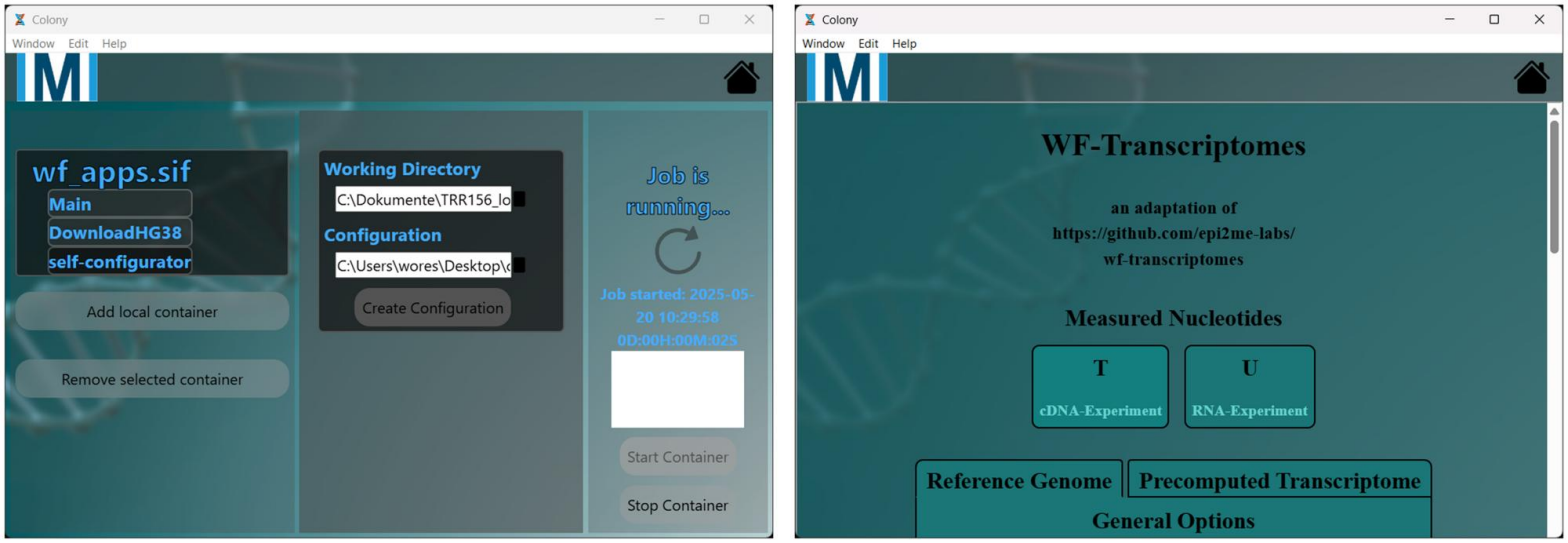
Two main pages encountered while working with the Colony launcher. Left: The container start page. Operating a container in itself is very simple and requires very few components. Right: A container-internal graphical user interface displayed within our tool. Application complexity is managed by the application itself.

We have created a tool that can be used to perform analyses with arbitrary Singularity containers. It supports containerized software that allows researchers to perform analyses on RNA-sequencing data in a portable and reproducible way. It comes with a graphical user interface that provides additional features to containers that are designed to use them but can be replaced by any web browser (Fig. 2). It guides the user and helps them collect and specify all necessary information for the workflow to run into a single configuration file. Software and configuration file can be stored and shared alongside all other research data, which increase the quality of FAIR-compliance regarding software. The container and the used configuration file can be exported with all other input data into a local repository. This repository can be moved over to remote systems manually, e.g. HPCs, and the analysis can be run there unchanged.

## 4 Discussion

We developed the framework since preexisting solutions, while numerous and mature, did not meet all of our requirements. EPI2ME Desktop, from which we drew great inspiration, is cost-free software but not open-source and might change its cost structure in the future. It not only uses open-source Nextflow workflows for analyses but also reduces the researchers’ control over their setup. For instance, updates may prevent previous versions of workflows from running. While it is possible to work around this issue, users need to exit the GUI and perform several complex steps including trial-and-error version compatibility checks between Nextflow and the workflow itself. All components to run the workflow exist and they are accessible, but the interplay between the components hinders their usage and reproducibility. In our framework, we follow a different strategy and limit the number of dependencies to Singularity and the container itself. The container can be used over the command line, standalone, with a standardized oneliner. In contrast to workflow managers like Snakemake and Nextflow, application complexity is not exposed to the user on the command line but captured inside a configuration file.

This approach makes Colony’s reproducibility essentially maintenance-free, since containers encapsulate all required functionality. Existing alternatives are often partially containerized only and rely on user-level applications, that may undergo changes and require each individual workflow to be adapted. Due to lazy loading of dependencies, workflows may become inaccessible over time. By allowing flexible web interfaces inside containers, Colony preserves user interactivity without compromising reproducibility.

Colony’s export feature further simplifies workflow execution. While execution on HPC systems requires manual transfer of the container and configuration, this process is comparable in complexity to alternatives such as Snakemake or Nextflow, which also require cluster-specific setup, scripting, or workarounds in cases like interactive authentication. Exporting the container allows non-expert users to avoid repetitive, error-prone steps. Once exported, the workflow invocation remains consistent, reducing mental load and supporting reusability. This export feature simplifies depositing software pipelines and entire experiments including raw data in repositories such as Zenodo or Harvard Dataverse, which ensures persistent identifiers and long-term accessibility in line with the FAIR principles. Colony’s strategy around containerization allows us to handle edge-cases such as non-workflow binaries and license-limited software, which we may modify and redistribute but only within our own organization as internal use case. Thus, we are able to apply FAIRness to software even in constrained settings.

Colony relies on Singularity rather than, e.g. Docker for containerization for portability reasons. Firstly, Singularity does not require root permissions. Secondly, a Singularity container is stateless and therefore compatible with parallel filesystems that are common on HPC systems. Docker containers are typically incompatible with HPC systems. By choosing Singularity containerization as the single uppermost software layer for core functionality, Colony inherits its great potential to conserve research software over time. Our framework simplifies the usage of software containers and might extend the adoption of containerized software applications, and in turn improve the quality of FAIR compliance regarding software.

## Data Availability

Windows Installer, container and example analysis are available on Harvard Dataverse, at https://dx.doi.org/10.7910/DVN/Z3OTWY.
